# Spatial and structural characteristics of occupied nests of *Calonectris borealis* (Cory, 1881) in the Central Azores in 2025

**DOI:** 10.3897/BDJ.14.e191989

**Published:** 2026-05-18

**Authors:** Laurine M. Parmentier, Alice Vedovelli, Andrea Petrone, Rúben MV Coelho, Miriam C. García, Guilherme Oyarzabal, Verónica Neves, Paulo A.V. Borges

**Affiliations:** 1 University of Azores, CE3C—Centre for Ecology, Evolution and Environmental Changes, Azorean Biodiversity Group, CHANGE —Global Change and Sustainability Institute, School of Agricultural and Environmental Sciences, Rua Capitão João d’Ávila, Pico da Urze, 9700-042, Angra do Heroísmo, Azores, Portugal University of Azores, CE3C—Centre for Ecology, Evolution and Environmental Changes, Azorean Biodiversity Group, CHANGE —Global Change and Sustainability Institute, School of Agricultural and Environmental Sciences, Rua Capitão João d’Ávila, Pico da Urze, 9700-042 Angra do Heroísmo, Azores Portugal; 2 University of Sassari, Piazza Università, 21, 07100, Sassari, Italy University of Sassari, Piazza Università, 21, 07100 Sassari Italy; 3 University of Bologna, via Zamboni 33, 40126, Bologna, Italy University of Bologna, via Zamboni 33, 40126 Bologna Italy; 4 Serviço de Ambiente da Terceira, Rua do Galo, nº 118, 9700-091, Angra do Heroísmo, Portugal Serviço de Ambiente da Terceira, Rua do Galo, nº 118, 9700-091 Angra do Heroísmo Portugal; 5 Regional Directorate for Maritime Policies, 29 Rua D. Pedro IV, Horta, Portugal Regional Directorate for Maritime Policies, 29 Rua D. Pedro IV Horta Portugal; 6 OKEANOS - Institute of Marine Sciences, University of the Azores, Horta, Azores, Portugal OKEANOS - Institute of Marine Sciences, University of the Azores Horta, Azores Portugal; 7 IUCN SSC Monitoring Specialist Group, Angra do Heroísmo, Azores, Portugal IUCN SSC Monitoring Specialist Group Angra do Heroísmo, Azores Portugal

**Keywords:** Cory's shearwater, seabird conservation, vegetation survey, Macaronesia, Darwin Core, seabird microhabitat, Braun-Blanquet

## Abstract

**Background:**

The Azores Archipelago hosts approximately 75% of the global breeding population of Cory’s shearwater (*Calonectris borealis*). This species faces threats ranging from habitat degradation and invasive predators to climate change, which increase the vulnerability of its breeding sites. Effective conservation and threat assessment require data on breeding habitats, yet such information remains scarce. To address this gap, we aimed to provide a standardised baseline of the spatial and structural characteristics of active nests in the Azores Region.

**New information:**

We present a comprehensive dataset based on a field survey conducted in 2025 across 11 colonies on four islands of the Azorean Central Group (Faial, Graciosa, Pico and Terceira). The dataset characterises 421 occupied nests and includes 5,812 individual records describing nest architecture (e.g. depth, width and substrate) and local topography (e.g. slope, orientation and proximity to the coast and infrastructure). Additionally, it contains 1,380 botanical records from vegetation surveys, identifying 106 vascular plant species. All data are standardised according to Darwin Core (DwC) requirements and are publicly available through the Global Biodiversity Information Facility (GBIF). This data package provides a resource for future spatial modelling and the development of targeted conservation strategies for seabirds.

## Introduction

Despite their role as bioindicators, top predators and nutrient vectors (Otero et al. 2018, Hazen et al. 2019), seabirds represent one of the most threatened avian groups globally (Dias et al. 2019). They are exposed to intensifying environmental challenges, especially marine birds of the order Procellariiformes, such as Cory’s shearwater (Sydeman et al. 2012, Dias et al. 2019, Afonso et al. 2020). These pressures include plastic pollution (Browne et al. 2011, Amélineau et al. 2016), bycatch (Da Rocha et al. 2021, Phillips et al. 2023), invasive species (Courchamp et al. 2007, Phillips et al. 2023), habitat degradation (Trathan et al. 2014, Kavelaars et al. 2020, Salas et al. 2020) and the impacts of climate change, including rising temperatures (Croxall et al. 2012, Keogan et al. 2018).

The Azores Archipelago is a critical region for seabird conservation, hosting significant breeding populations of various species (Afonso et al. 2020). Most notably, it is a major nesting area for *C. borealis*, with around 75% of the global breeding population nesting within the Archipelago (BirdLife International 2018, Afonso et al. 2020). This represents around 180,000 breeding pairs returning each year to the Azores to breed in small cavities known as burrows or nesting burrows (called nest hereafter to avoid confusion) (Monteiro et al. 1996, BirdLife International et al. 2004). Due to its high numbers and importance, this species is the focus of intense conservation efforts, such as the annual rescue campaign SOS Cagarro and legal protections from the European Union’s Birds Directive (European Parliament and Council 2009) and the local Regional Regulatory Decree (Diário da República 2020).

Several factors make *C. borealis* vulnerable in its breeding colonies. As females lay a single egg per year resulting in a maximum of one fledgling (Monteiro et al. 1996), the species has a low reproductive rate and, therefore, its populations are vulnerable to stressors. In the Azores, these birds face challenges, such as breeding habitat vulnerability (Ng et al. 2019), with exposure to rising temperatures and increasing extreme weather events (Dias et al. 2019) and predation by invasive species (Lamelas-Lopez et al. 2023).

Recent research has highlighted that nest-specific characteristics are critical determinants of breeding success and predator vulnerability (Rodríguez et al. 2021). Furthermore, as heat stress becomes a growing threat, the physical characteristics of the nest are useful for assessing whether they can provide a thermal refuge for the chicks (Oswald and Arnold 2012, Richards et al. 2024). Beyond that, the surrounding vegetation plays a role in shaping the nesting microhabitat. Plant communities contribute to soil stability (Pérès et al. 2013, Gyssels et al. 2024), which is vital for the structural integrity of nests. Furthermore, vegetation acts as a biological buffer, offering shade and providing protection from predators (Wolf et al. 1996).

Despite the clear link between habitat features and breeding success, high-resolution and standardised datasets with these parameters remain scarce across the Azores. We provide a standardised dataset designed for long-term reuse and open access. This information serves as a baseline for spatial habitat modelling and threat assessments, ultimately supporting evidence-based conservation for Azorean seabird colonies.

## General description

### Purpose

The purpose of this dataset is to provide high-resolution spatial and structural data for 421 active Cory’s shearwater nests. This baseline information is intended to: 1) address the current knowledge gap; and 2) support future studies and evidence-based conservation actions across the Azores Central Group.

## Project description

### Title

Spatial and structural characteristics of occupied nests of *Calonectris borealis* (Cory, 1881) in the Central Azores in 2025

### Personnel

Laurine M. Parmentier, Alice Vedovelli, Andrea Petrone, Rúben Coelho, Miriam C. Garcia, Amelie Neitzel, Ambre Solivellas, Guilherme Oyarzabal, Verónica Neves, Paulo A.V. Borges. Fieldwork licence n.º 57/2025/DRAAC.

### Study area description

**Figure 1.**
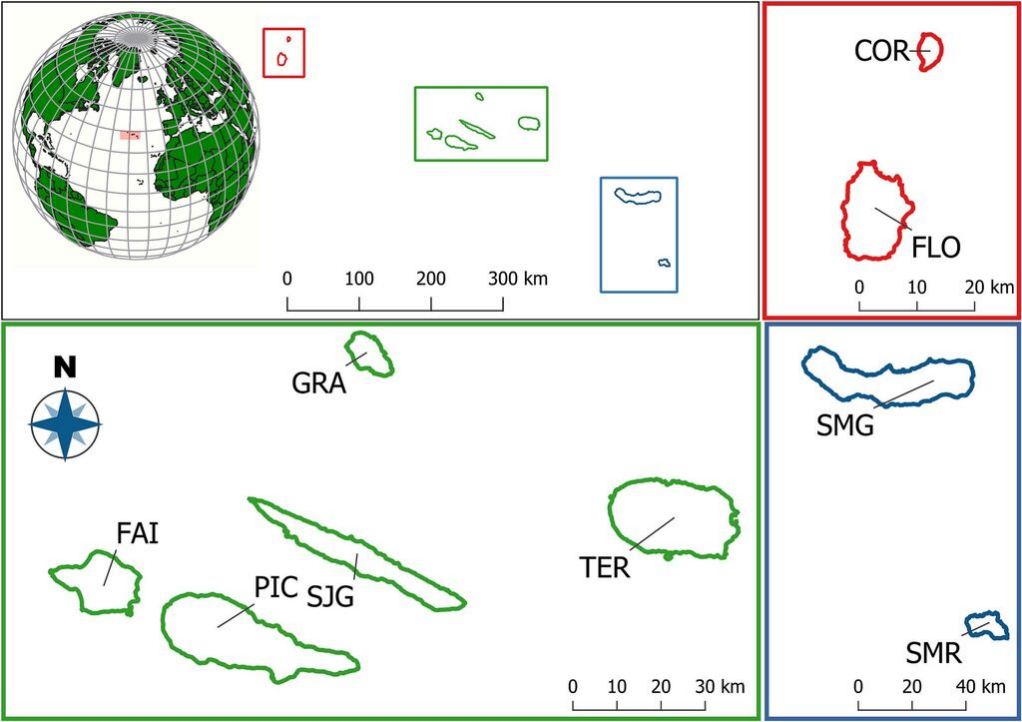
Map of the Azores Archipelago. Top left shows the position of the three island groups: Western group – red for Corvo (COR) and Flores (FLO); Central group – green for Faial (FAI), Pico (PIC), São Jorge (SJG), Graciosa (GRA) and Terceira (TER); and Eastern group – blue for São Miguel (SMG) and Santa Maria (SMR). Adapted from Oyarzabal et al. (2025).

We conducted this study across 11 colonies of Cory's shearwater, located on four islands within the Central Group of the Azores Archipelago (North Atlantic) (Fig. 1). The islands included in this study are Graciosa (39°3′5″N 28°0′51″W), Terceira (38°43′40″N 27°12′48″W), Pico (38°27′57″N 28°20′0″W) and Faial (38°34′57″N 28°42′17″W).

**Figure 2.**
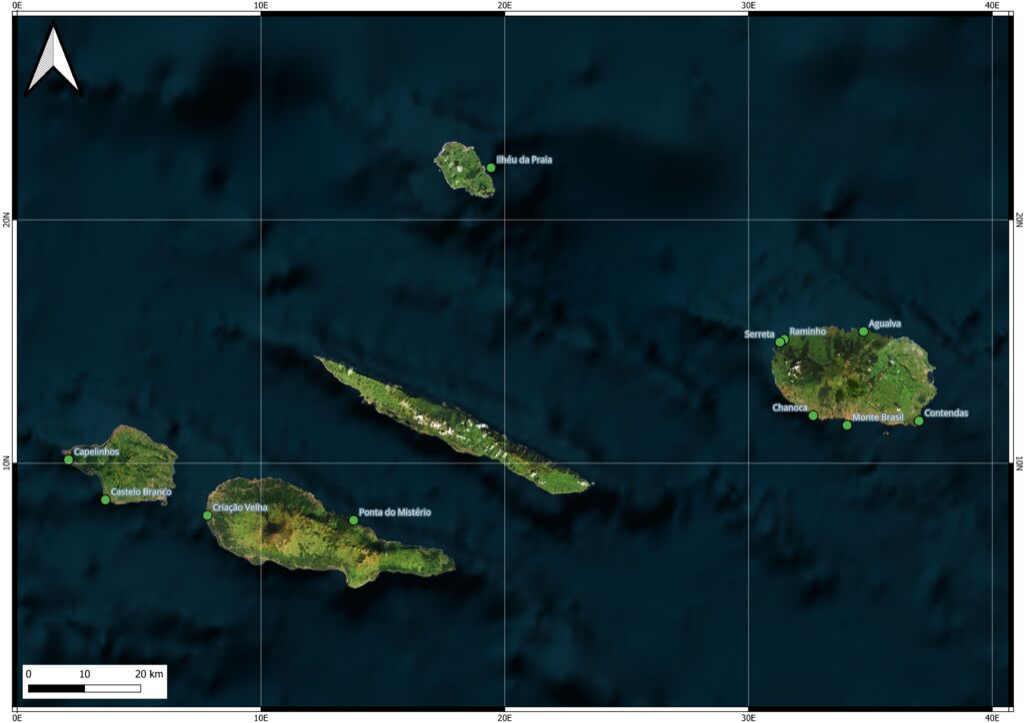
Location of the 11 *C. borealis* colonies surveyed in the Azorean Central Group during the breeding season of 2025. The islands shown include Faial (colonies of Capelinhos and Castelo Branco), Graciosa (colony of Ilhéu da Praia), Pico (colonies of Criação Velha and Ponta do Mistério) and Terceira (colonies of Monte Brasil, Chanoca, Serreta, Raminho, Agualva and Contendas).

Sampling took place in 11 colonies (Fig. 2). On Graciosa, the Ilhéu da Praia colony is a rocky islet with vegetated lowlands. Terceira surveys included northern sites with cliffs covered by *Erica azorica* Hochst. ex Seub and *Morella faya* (Aiton) Wilbur (Agualva, Raminho, Serreta) and southern sites ranging from *Pittosporum undulatum* Vent. woodland (Monte Brasil) and vegetated hills (Contendas) to rocky bays with *Carpobrotus edulis* (L.) N.E.Br. (Chanoca). Pico sampling focused on the fragmented stone walls of the Criação Velha colony (also referred as Monte in other publications) and the *P. undulatum* boulders of Ponta do Mistério colony (also referred as Mistério da Prainha in other publications). Finally, Faial colonies comprised the rocky shoreline of the Capelinhos colony and the shrubby *E. azorica* cliffs of the Castelo Branco colony (referred as Castelo Branco hereafter).

### Design description

We selected the colonies and initial nests, based on historical data from the University of the Azores. We conducted systematic searches for additional active nests once in the breeding season. Due to the rugged volcanic topography of the islands, sampling was restricted to the accessible portions of each colony. Consequently, the dataset may reflect a spatial bias towards nests located on less steep or more stable terrain.

To locate new nests, we performed a systematic search once in the breeding season by walking through the accessible parts of the colonies and checking for different signs (vocalisations, presence of feathers, excrement or recently excavated soil). We confirmed occupancy by observing either an adult Cory's shearwater, an egg or a chick inside the nest. Each occupied nest was assigned a unique identifier, was physically marked and its precise geographic coordinates were mapped. The final dataset includes active nests that were found between April 2025 and October 2025.

### Funding

Research project funded by the Regional Science and Technology Fund (FRCT), Government of the Açores, Doctoral Scholarship M3.1.a/F/013/2024 (Programa PRO-SCIENTIA). A part of the fieldwork was funded by CE3C - Centre for Ecology, Evolution and Environmental Changes via a PhD student budget (Ref: UID/00329/2025; DOI: https://doi.org/10.54499/UID/00329/2025).

## Sampling methods

### Sampling description

We collected the data from April to October 2025 across 421 active nests, on the islands of Graciosa, Pico, Faial and Terceira. We used two types of survey and standardised field equipment.

Nest Characteristics Surveys (mapped to measurementOrFact extension) included physical dimensions (depth and width of the cavity, height and width of the entrance) measured in centimetres at the widest points. Nest orientation (compass) and slope (clinometer) were recorded in degrees ("measurementRemarks" column, measurementOrFact extension) and in radians ("measurementValue" column, measurementOrFact extension). Slope was measured as the terrain inclination outside the nest entrance, with positive values indicating a downward slope away from the entrance and negative values indicating a nest located within a depression. Precise coordinates and altitude were obtained via Garmin GPSmap 62s and Gaia GPS (Outside Interactive 2025). Distances to the ocean, roads and villages were calculated in QGIS using the Distance to nearest hub tool against point layers generated via Extract Vertices. These points were derived from a DEM-based binary sea mask and infrastructure data sourced via the QuickOSM plugin. Qualitative traits (nest type, substrate, openings, obstacles) were recorded via direct observation.

Vegetation Surveys (mapped to occurrence extension) were conducted in 2 m x 2 m plots centred on nest entrances (Chytrý and Otýpková 2003). Vascular plant taxa were identified and their cover-abundance was estimated using the Braun-Blanquet scale (r, +, 1, 2, 3, 4 and 5). All data collection followed standardised protocols and equipment to ensure cross-island consistency.

### Quality control

Data consistency was maintained via standardised protocols, team training and use of the same equipment across all islands. Nest coordinates were verified via GIS. LMP, AV and AP consistently applied Braun-Blanquet cover-abundance scores at all sites. AP performed or verified all field identifications. Species taxonomy and conservation status were assigned using the Azores Bioportal – PORBIOTA and the region's official biodiversity platform Flora-On.

### Step description

The 11 sites were identified via historical coordinates and additional systematic surveys. We confirmed occupancy with a direct observation of an adult, egg or chick. For each of the 421 occupied nests, we conducted standardised measurements and vegetation surveys using the Braun-Blanquet scale. Data were digitised and formatted according to Darwin Core (DwC) standards. In total, the database integrates 421 unique Event records (nests) linked to 1,223 records in the occurrence table and 5,812 records in the MeasurementOrFact table.

## Geographic coverage

### Description

The study was carried in 11 colonies in the islands of Faial, Graciosa, Pico and Terceira.

### Coordinates

38.492 and 39.058 Latitude; -28.818 and -27.077 Longitude.

## Taxonomic coverage

### Description

The following vascular plants orders are covered in the dataset "occurrence":

### Taxa included

**Table:** 

Rank	Scientific Name	
order	Alismatales	
order	Apiales	
order	Asterales	
order	Boraginales	
order	Brassicales	
order	Caryophyllales	
order	Commelinales	
order	Ericales	
order	Fabales	
order	Fagales	
order	Gentianales	
order	Lamiales	
order	Laurales	
order	Malpighiales	
order	Malvales	
order	Myrtales	
order	Oxalidales	
order	Poales	
order	Polypodiales	
order	Ranunculales	
order	Rosales	
order	Saxifragales	
order	Solanales	

## Temporal coverage

**Data range:** 2025-4-30 – 2025-10-01.

## Usage licence

### Usage licence

Creative Commons Public Domain Waiver (CC-Zero)

## Data resources

### Data package title

Characteristics of occupied nests Cory’s shearwater *Calonectris borealis* (Cory, 1881) in the Central Group of islands, Azores, during the breeding season 2025

### Resource link

https://doi.org/10.15468/h2p6qk

### Alternative identifiers

https://ipt.gbif.pt/ipt/resource?r=characteristicsnests_coryshearwater_2025; https://www.gbif.org/dataset/9aa6ca9f-1596-47bd-a51e-ffc8b3986da1

### Number of data sets

3

### Data set 1.

#### Data set name

Event table

#### Data format

Darwin Core Archive

#### Character set

UTF-8

#### Download URL

https://ipt.gbif.pt/ipt/resource?r=characteristicsnests_coryshearwater_2025

#### Data format version

1.6

#### Description

The dataset was published in the Global Biodiversity Information Facility platform (GBIF) (Parmentier et al. 2026). The following data table includes the records for which a taxonomic identification of the species was possible. The dataset submitted to GBIF is structured as an event table with data on nest locations, that has been published as a Darwin Core Archive (DwCA), which is a standardised format for sharing biodiversity data as a set of one or more data tables. The core data file contains 421 event records (eventID). The dataset was archived using the GBIF Integrated Publishing Toolkit (IPT, version 2.5.6), which serves as the data repository. The data and associated resource metadata are available for download through the Portuguese GBIF Portal IPT (Parmentier et al. 2026).

**Data set 1.** 

Column label	Column description
eventID	Identifier of the events, unique for the dataset.
basisOfRecord	Specific nature of the data record.
samplingProtocol	Sampling method used to obtain the records.
eventDate	Date or date range the record was collected.
day	Day of the event.
month	Month of the event.
year	Year of the event.
stateProvince	Region of the sampling site.
islandGroup	Name of the archipelago of the sampling site.
island	Name of the island of the sampling site.
country	Name of the country of the sampling site.
countryCode	ISO code of the country of the sampling site.
municipality	Full name of the municipality of the sampling site.
locality	Name of the locality of the sampling site.
minimumElevationInMetres	Minimum elevation of the sampling site in metres above sea level.
locationRemarks	Remarks about the location of the sampling site.
decimalLatitude	Geographic latitude, in decimal degrees.
decimalLongitude	Geographic longitude, in decimal degrees.
geodeticDatum	Ellipsoid, geodetic datum or spatial reference system (SRS) upon which thegeographic coordinates given in decimalLatitude and decimalLongitude are based.
coordinateUncertaintyInMetres	Uncertainty of the coordinates, in metres.
coordinatePrecision	Precision of the coordinates.
georeferenceSource	List (concatenated and separated) of maps, gazetteers or other resources used to georeference the Location, described specifically enough to allow anyone in the future to use the same resources.

### Data set 2.

#### Data set name

Measurement or Fact

#### Data format

Darwin Core Archive

#### Character set

UTF-8

#### Download URL

https://ipt.gbif.pt/ipt/resource?r=characteristicsnests_coryshearwater_2025

#### Data format version

1.6

#### Description

The dataset was published in the GBIF platform (Parmentier et al. 2026). In addition to the core event and occurrence tables, the dataset includes a **MeasurementOrFact** extension table within the same DwCA. This extension compiles all quantitative and qualitative attributes associated with the occurrence records (linked via **occurrenceID**), storing each observation/measurement as a separate row (i.e. one occurrence can have multiple measurements). The MeasurementOrFact table, therefore, provides the structured repository for ancillary information recorded in the field complementing the taxonomic and spatiotemporal information stored in the occurrence core. This DwCA was generated and archived using the GBIF Integrated Publishing Toolkit (IPT, Version 2.5.6), which serves as the data repository. The full dataset (core and two extensions) and its metadata are available for download from the Portuguese GBIF Portal IPT (Parmentier et al. 2026). The measurements or facts give information about different numerical and categorical variables measured either with adequate tools and software or with observations made on-site, as described in the sampling methods.

**Data set 2.** 

Column label	Column description
eventID	Identifier of the events, unique for the dataset.
measurementID	Identifier of the measurements, facts, characteristics or assertions observed, unique for the dataset.
institutionID	Identity of the institution publishing the data.
institutionCode	Code of the institution publishing the data package.
measurementType	Nature of the measurement, fact, characteristic or assertion.
verbatimMeasurementType	String representing the type of measurement or fact as it appeared in the original record.
measurementValue	Value of the measurement, fact, characteristic or assertion.
measurementAccuracy	Accuracy of the measurement or fact value.
measurementUnit	Unit associated with the measurement or fact value. In this dataset, depending on the measurement or fact, the unit be metres, centimetres, radians and degrees or an integer number.
measurementDeterminedBy	List (concatenated and separated) of names of people, groups or organisations who determined the value of the measurement or fact.
measurementDeterminedDate	Date on which the measurement or fact was made.
measurementMethod	Description of or reference to (publication, URI) the method or protocol used to determine the measurement, fact, characteristic or assertion.
measurementRemarks	Comment accompanying the measurement or fact.

### Data set 3.

#### Data set name

Occurrence

#### Data format

Darwin Core Archive

#### Character set

UTF-8

#### Download URL

https://ipt.gbif.pt/ipt/resource?r=characteristicsnests_coryshearwater_2025

#### Data format version

1.6

#### Description

The dataset was published in the GBIF plateform (Parmentier et al. 2026). The following data table includes all the records for which a taxonomic identification of the species was possible. The dataset submitted to GBIF is structured as an occurrence table that has been published as a DwCA, which is a standardised format for sharing biodiversity data as a set of one or more data tables. The core data file contains 1223 records (occurrenceID). This GBIF IPT (Integrated Publishing Toolkit, Version 2.5.6) archives the data and, thus, serves as the data repository. The data and resource metadata are available for download in the Portuguese GBIF Portal IPT (Parmentier et al. 2026).

**Data set 3.** 

Column label	Column description
eventID	Identifier of the events, unique for the dataset.
occurrenceID	Identifier of the record, coded as a global unique identifier.
institutionID	Identity of the institution publishing the data.
institutionCode	Code of the institution publishing the data package.
basisOfRecord	Nature of the recorded data.
scientificName	Complete scientific name of recorded species, including authorship and date information if known.
kingdom	Full scientific name of the kingdom of the recorded species.
phylum	Full scientific name of the phylum name of the recorded species.
class	Full scientific name of the class name of the recorded species.
order	Full scientific name of the order name of the recorded species.
family	Full scientific name of the family name of the recorded species.
subfamily	Full scientific name of the subfamily name of the recorded species.
genus	Full scientific name of the genus name of the recorded species.
specificEpithet	Full scientific name of the specific epithet of the recorded species.
infraspecificEpithet	Full scientific name of the infraspecific epithet of the recorded species.
scientificNameAuthorship	Authorship information of the lowest taxon rank included in the record, formatted according to the conventions of the applicable nomenclatural code.
establishmentMeans	Process of establishment of the species in the location, using a controlled vocabulary: 'native', 'introduced', 'endemic' or 'indeterminate'.
degreeOfEstablishment	Degree to which the species survives, reproduces and expands its range in the given location and time.
taxonRank	Taxonomic rank of the most specific name.
occurrenceStatus	Presence or absence of the taxon.
recordedBy	List (concatenated and separated) of names of people, groups or organisations who assigned the taxon to the record.
organismQuantity	Number or enumeration value for the quantity of organisms.
organismQuantityType	Type of quantification system used for the quantity of organisms.

## Additional information

### Multiple occupancy

In Pico (Criação Velha and Ponta do Mistério colonies), several lava tubes function as cavities hosting multiple breeding pairs (nests: CV005, CV006, CV040, CV065, PM057). In the dataset, these are identified by a shared **eventID** with alphabetical suffixes (e.g. CV005A, CV005B) to distinguish individual nests. Each suffix corresponds to independent records in the *Event*, *MeasurementOrFact* and *Occurrence* tables, ensuring data integrity for each nest despite the shared geological structure.

### Nest characteristics

Table 1 below summarises the mean, median and range of each variable by island. Slope and orientation are presented in degrees for common usage.

**Figure 3.**
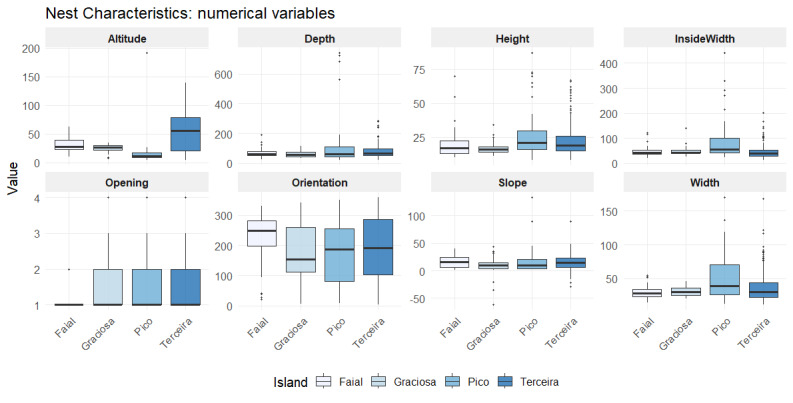
Boxplots representing the distribution of nest charactristics and topographic variables of *C. borealis* across Faial, Graciosa, Pico and Terceira. Numerical variables shown include nest depth, height, width, inside width (all in cm), altitude (m) and terrain slope and entrance orientation (degrees). Credits: LMP.

**Figure 4.**
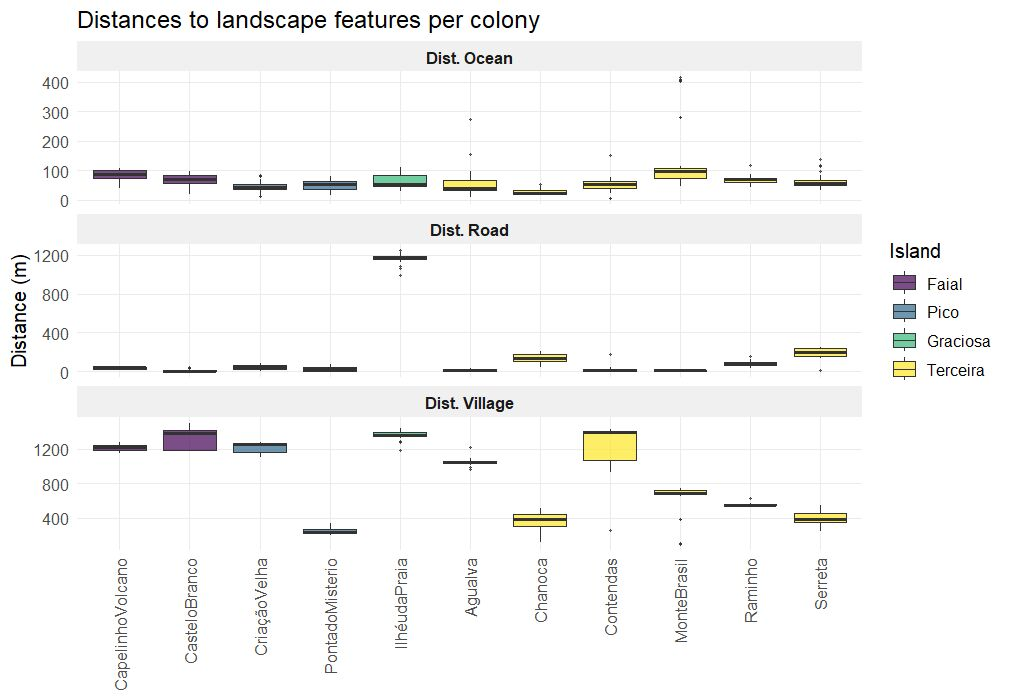
Boxplots showing the distances in metres between *C. borealis* nests and the ocean, the nearest road and the nearest village for all surveyed colonies in the Central Group. Credits: LMP.

The following figures (Figs 3, 4) summarise the numerical variables that were recorded, with one boxplot per island. The boxplots are showing the distribution of structure nest variables (Fig. 3) and spatial variables (Fig. 4).

**Figure 5.**
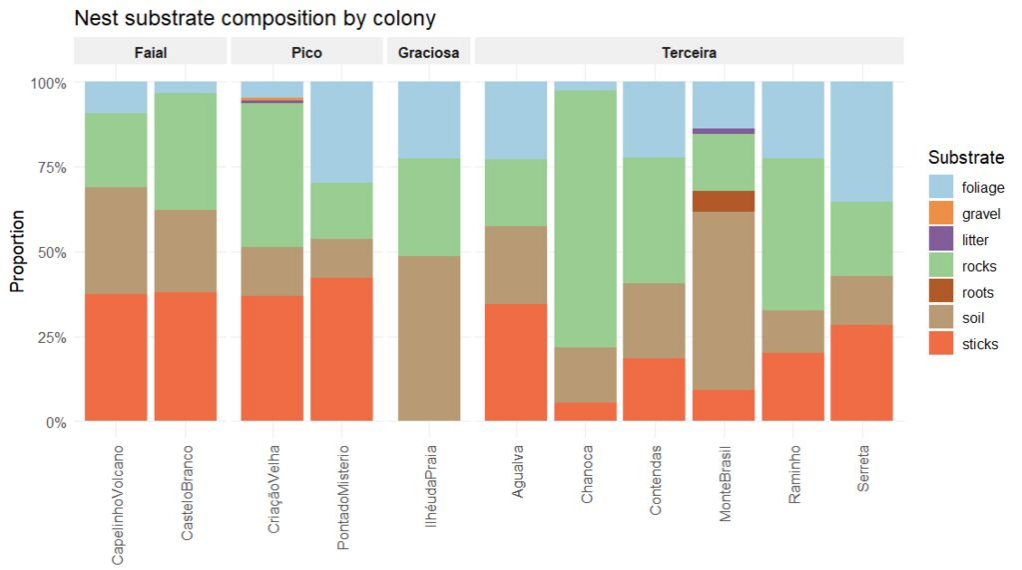
Proportion of substrate types found inside the nests in the studied colonies across the islands of Faial, Graciosa, Pico and Terceira. Credits: LMP.

**Figure 6.**
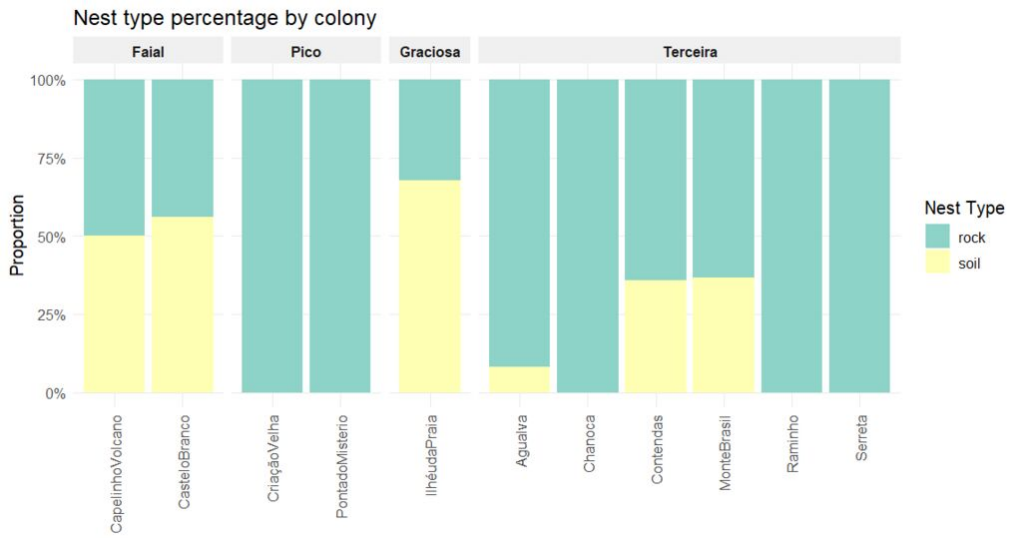
Proportion of the nest type in the studied colonies across the islands of Faial, Graciosa, Pico and Terceira. Credits: LMP.

Fig. 5 shows the different materials that constitute the nest substrate per colony. They are soil-based, rocks and foliage across all sites, with sticks present everywhere, except Graciosa. Restricted materials include litter (Monte Brasil and Criação Velha) and gravel (Criação Velha). Nest type (Fig. 6) shows the percentage of each nest type per colony. Five colonies display only one type of nest.

### Vegetation survey

The tables (Tables 2, 3) summarise frequent vascular plants at island and colony scales. Island-level dominance (Table 2) includes endemic *Erica azorica* Hochst. ex Seub. (Terceira, Pico) and Daucus carota subsp. azoricus Franco (Faial, Graciosa), alongside invasive *Pittosporum undulatum* Vent. (Pico) and *Carpobrotus edulis* (L.) N.E.Br. (Faial) and *Portulaca oleracea* L. (Graciosa). Colony-specific data (Table 3) shows shifts between endemic and invasive dominance.

### Limitations

Sampling effort was higher on Terceira due to logistical constraints and the exclusion of inaccessible nests for safety reasons.

## Figures and Tables

**Table 1.** Summary statistics (mean, median, minimum and maximum) of all the variables from the Nest Characteristics Survey for Cory’s shearwater (*Calonectris borealis*) across four islands in the Central Group of the Azores, Faial, Graciosa, Pico and Terceira (cm: centimetres; m: metres; m. a. s. l.: metres above sea level).

**Island**	**Variable**	**Mean**	**Median**	**Min**	**Max**
Faial	Altitude (m. a. s. l.)	31.59	28	10	63
Faial	Depth (cm)	70.35	63	24	192
Faial	DistOcean (m)	76.98	76.91	20.01	109.43
Faial	DistRoad (m)	25.99	29.72	0.86	50.4
Faial	DistVillage (m)	1255.17	1233.66	1154.47	1495.33
Faial	Height (cm)	19.4	17	10	70
Faial	InsideWidth (cm)	46.9	42	22	121
Faial	Openings (integer)	1.18	1	1	2
Faial	Orientation (degrees)	228.3	250	21	330
Faial	Slope (degrees)	16.62	16	1	40
Faial	Width (cm)	29.51	28	14	55
Graciosa	Altitude (m. a. s. l.)	24.94	27	8	35
Graciosa	Depth (cm)	59.76	56.5	35	117
Graciosa	DistOcean (m)	63.82	54.32	30.89	110.23
Graciosa	DistRoad (m)	1171.47	1171.34	988.48	1247.75
Graciosa	DistVillage (m)	1363.86	1359.18	1188.13	1437.04
Graciosa	Height (cm)	17.18	16	11	34
Graciosa	InsideWidth (cm)	50.09	43	27	140
Graciosa	Openings (integer)	1.47	1	1	4
Graciosa	Orientation (degrees)	179.56	153.5	5	342
Graciosa	Slope (degrees)	8.76	9.5	-62	44
Graciosa	Width (cm)	30.56	30	20	46
Pico	Altitude (m. a. s. l.)	14.68	12	4	191
Pico	Depth (cm)	166.91	62	21	740
Pico	DistOcean (m)	46.82	44.23	11.87	84.09
Pico	DistRoad (m)	38.25	40.79	0.68	83.71
Pico	DistVillage (m)	840.66	1149.82	201.28	1281.66
Pico	Height (cm)	30.66	21	8	87
Pico	InsideWidth (cm)	106.24	56	24	440
Pico	Openings (integer)	1.41	1	1	4
Pico	Orientation (degrees)	176.31	187	7	350
Pico	Slope (degrees)	15.31	10	2	90
Pico	Width (cm)	50.13	39	12	170
Terceira	Altitude (m. a. s. l.)	52.76	55	5	139
Terceira	Depth (cm)	80.47	68	20	286
Terceira	DistOcean (m)	70.16	57.22	4.79	414.29
Terceira	DistRoad (m)	92.77	69.53	0.2	251.93
Terceira	DistVillage (m)	636.28	546.5	96.69	1422.14
Terceira	Height (cm)	22.66	19	8	67
Terceira	InsideWidth (cm)	44.87	40	12	202
Terceira	Openings (integer)	1.43	1	1	4
Terceira	Orientation (degrees)	191.03	192	3	359
Terceira	Slope (degrees)	16.8	14	-29	90
Terceira	Width (cm)	36.86	30	11	168

**Table 2.** Most frequently occurring plant species identified during the vegetation survey in the islands of Faial, Graciosa, Pico and Terceira.

**Island**	**Scientific Name**	**Occurrence Count**
Faial	*Carpobrotus edulis* (L.) N.E.Br.	35
Faial	Daucus carota subsp. azoricus Franco	32
Faial	*Pteridium aquilinum* (L.) Kuhn	27
Graciosa	*Portulaca oleracea* L.	19
Graciosa	*Brachypodium sylvaticum* (Huds.) P.Beauv.	17
Graciosa	Daucus carota subsp. azoricus Franco	13
Pico	*Pittosporum undulatum* Vent.	49
Pico	*Erica azorica* Hochst. ex Seub.	37
Pico	Daucus carota subsp. azoricus Franco	35
Terceira	*Erica azorica* Hochst. ex Seub.	100
Terceira	*Morella faya* (Aiton) Wilbur	70
Terceira	*Pittosporum undulatum* Vent.	52

**Table 3.** The most frequently occurring plant species identified in the vegetation plots surrounding the occupied nests across the eleven colonies.

**Colony**	**Island**	**Scientific Name**	**Occurrence Count**
Capelinhos	Faial	*Carpobrotus edulis* (L.) N.E.Br.	32
Castelo Branco	Faial	*Tetragonia tetragonoides* (Pall.) Kuntze	18
Ilhéu da Praia	Graciosa	*Portulaca oleracea* L.	19
Criação Velha	Pico	Daucus carota subsp. azoricus Franco	35
Mistérios da Prainha	Pico	*Pittosporum undulatum* Vent.	46
Agualva	Terceira	*Erica azorica* Hochst. ex Seub.	28
Monte Brasil	Terceira	*Pittosporum undulatum* Vent.	34
Raminho	Terceira	*Erica azorica* Hochst. ex Seub.	21
Serreta	Terceira	*Morella faya* (Aiton) Wilbur	54
Chanoca	Terceira	*Carpobrotus edulis* (L.) N.E.Br.	10
Contendas	Terceira	*Pittosporum undulatum* Vent.	7
